# Simulated microgravity with floating environment promotes migration of non-small cell lung cancers

**DOI:** 10.1038/s41598-019-50736-6

**Published:** 2019-10-10

**Authors:** Chi Bum Ahn, Ji-Hyun Lee, Dae Geun Han, Hyun-Wook Kang, Sung-Ho Lee, Jae-Ik Lee, Kuk Hui Son, Jin Woo Lee

**Affiliations:** 1 Department of Molecular Medicine, College of Medicine, Gachon University, Incheon, Republic of Korea; 2 Department of Health Sciences and Technology, GAIHST, Gachon University, Incheon, Republic of Korea; 3 Department of Biomedical Engineering, School of Life Sciences, Ulsan National Institute of Science and Technology, Ulsan, Republic of Korea; 4 Department of Thoracic and Cardiovascular Surgery, Korea University Medical College, Korea University, Seoul, Republic of Korea; 5 Department of Thoracic and Cardiovascular Surgery, Gachon University Gil Medical Center, School of Medicine, Gachon University, Incheon, Republic of Korea

**Keywords:** Non-small-cell lung cancer, Astronomical instrumentation

## Abstract

A migration of cancer is one of the most important factors affecting cancer therapy. Particularly, a cancer migration study in a microgravity environment has gained attention as a tool for developing cancer therapy. In this study, we evaluated the proliferation and migration of two types (adenocarcinoma A549, squamous cell carcinoma H1703) of non-small cell lung cancers (NSCLC) in a floating environment with microgravity. When we measured proliferation of two NSCLCs in the microgravity (MG) and ground-gravity (CONT), although initial cell adhesion in MG was low, a normalized proliferation rate of A549 in MG was higher than that in CONT. Wound healing results of A549 and H1703 showed rapid recovery in MG; particularly, the migration rate of A549 was faster than that of H1703 both the normal and low proliferating conditions. Gene expression results showed that the microgravity accelerated the migration of NSCLC. Both A549 and H1703 in MG highly expressed the migration-related genes MMP-2, MMP-9, TIMP-1, and TIMP-2 compared to CONT at 24 h. Furthermore, analysis of MMP-2 protein synthesis revealed weaker metastatic performance of H1703 than that of A549. Therefore, the simulated microgravity based cancer culture environment will be a potential for migration and metastasis studies of lung cancers.

## Introduction

Lung cancer, which accounts for 19% of all cancers, shows a high mortality rate. In 2015, 8.8 million people died from cancer; the most common cause of cancer death was lung cancer, which caused 1.69 million deaths^1,2^. Additionally, in the United States, approximately 113,000 men and 103,000 women were diagnosed with lung cancer in 2014, and 156,000 people died from this disease^3^. Among patients diagnosed with lung cancer, only 21% do not show metastasis and 50% cannot undergo surgical operation because of cancer metastasis. A total of 70% of lung cancer patients die within one year and 87% die within five years. Because of the variety of lung cancers, patients often show different clinical conditions. Lung cancer is classified into two types: small cell lung cancer and non-small cell lung cancer (NSCLC); NSCLC accounts for over 85% of clinically diagnosed lung cancers. Types of NSCLC include lung adenocarcinoma, squamous cell carcinoma, and large cell carcinoma. They mutually vary in clinical characteristics such as easy sites, type, speed, and symptoms in different types of tissue-type and are treated using different methods^4^.

In the metastatic process, cancer cells form new colonies from their original sites to other locations, which involves multiple mechanisms. Metastasis is largely divided into two steps: obtaining the ability to move away from the primary site and proliferating at the second position. Metastatic potential depends on these two factors^5–7^. Particularly, metastasis is a very important factor in lung cancer and is associated with high death rates. As a result, many researchers have sought to discover the mechanism of cancer metastasis.

A consensus has emerged regarding the need to observe human body changes in a low- or zero-gravity environment. And when we focused on the each cell, a change in physical constraints such as the gravity causes to modulate phenotypic transitions occurring both in physiological setting and pathological setting^8^. However, because experiments in space are limited by a lack of opportunities and resources, a 2D clinostat and 3D clinostat known as a random positioning machine (RPM) were developed to mimic a micro- or near zero-gravity environment^9–12^. In a 2D clinostat, the object is rotated around one axis perpendicular to the force of the gravity and the maximal acceleration can be calculated by the diameter of rotation and the rotation speed^13–15^. In a RPM (3D clinostat), the object rotates around two independent axes in order to provide a status of “vector averaged gravity”. Because RPM can control the gravity applied to objects more precisely than 2D clinostat with a lower rotating speed, most of the recent studies has been progressed using RPM^16,17^. RPM produces an effect that resembles real microgravity because of the continuous change in the gravity vector and relative alignment position of the object when the device rotates faster than the time over which the object senses gravity. Additionally, to avoid centrifugal effects, the RPM rotates at a sufficiently small angular velocity, so that objects in the center of the RPM axis are placed without motion. Although RPMs cannot completely mimic the cosmic environment, simulated microgravity created by an RPM may be useful in cancer research, stem cell therapy, tissue engineering, and regenerative medicine.

A recent study reported that a microgravity environment caused by an RPM altered cancer metastasis^18^. Particularly, a microgravity environment altered the differentiation and increased the apoptosis of thyroid cancer cells. This hypothesis was confirmed by an experiment in the Shenzhou-8 space rocket^19^. After stimulating human breast carcinoma^20^, lung adenocarcinoma^21^, and human glioblastoma^22^ in the RPM, the proliferation and migration abilities of these cells and non-stimulated cancer cells were compared. The results indicated that cancer cell proliferation and metastasis were reduced following exposure to the RPM. However, the results were not observed in real-time. In previous studies, cancer cells were fixed to the culture plate by RPM and rotated along the axis and the culture media stimulated the attached cells through the rotational movement of the system^23^. This made it difficult to assess the effect of cells subjected to microgravity. Therefore, in this study, a culture membrane that was buoyant in the media was used to attach and culture lung cancer cells, enabling the cells to float in the media. After preparing a soft chamber of oxygen permeable material, culture media was filled in the chamber and packed with the culture membrane with the attached cells. In this study, we observed changes in the proliferation and migration of two different types of non-small cell lung cancers in a microgravity environment rather than after removing cells from microgravity stimulation.

## Results

### Effects of microgravity on lung cancer cell proliferation

**Figure 1:**
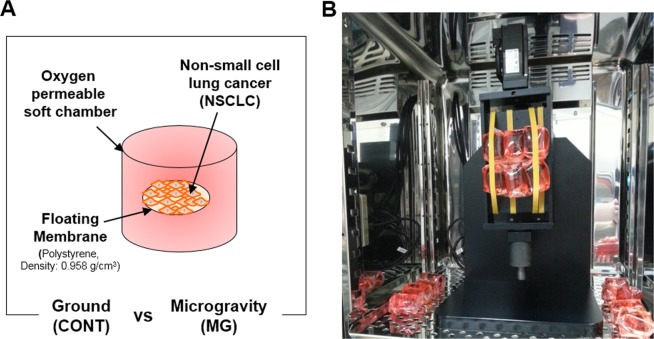
System configuration of simulated microgravity with a floating environment. Schematic diagram of non-small cell lung cancer (NSCLC) culture system (**A**). Installation of microgravity system in the CO_2_ incubator (**B**).

**Figure 2:**
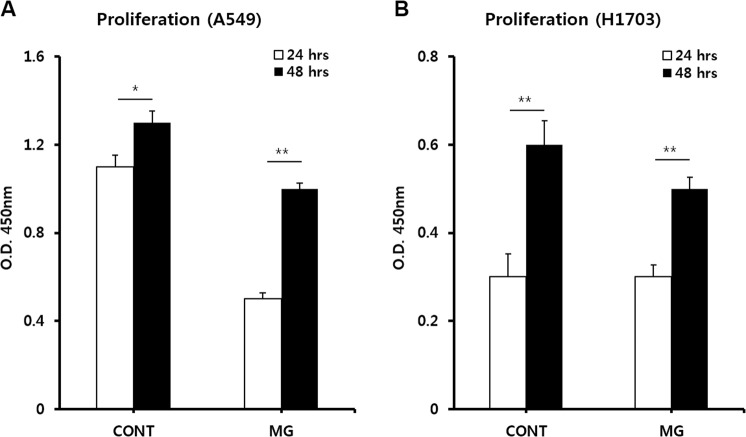
Proliferation analysis of A549 and H1703 cells. Comparison of A549 proliferation between ground (CONT) and microgravity (MG) (**A**). Comparison of H1703 proliferation between CONT and MG (**B**) (*p < 0.05, **p < 0.01).

To investigate whether microgravity affects the proliferation potential of lung cancer cells, cell viability tests were conducted. First, the MG and CONT groups were prepared and installed into the CO_2_ incubator (Fig. 1A,B). At 24 and 48 h after starting the experiment, A549 cells in both the CONT and MG showed increased cell numbers (Fig. 2A). A larger number of cells was attached to the membrane at 24 h in the CONT than in the MG. This is because the initial change in gravity by the spinning motion caused the seeded cells to be removed from the membrane. However, at 48 h, the rate of proliferation was higher in the MG than in the CONT. The proliferation rate of H1703 cells in the CONT was slightly higher than that in the MG at 24 and 48 h (Fig. 2B). Additionally, cell proliferation of A549 cells was faster than that of H1703 cells, despite initial cell seeding at the same level.

### Effects of microgravity on lung cancer cell migration

**Figure 3:**
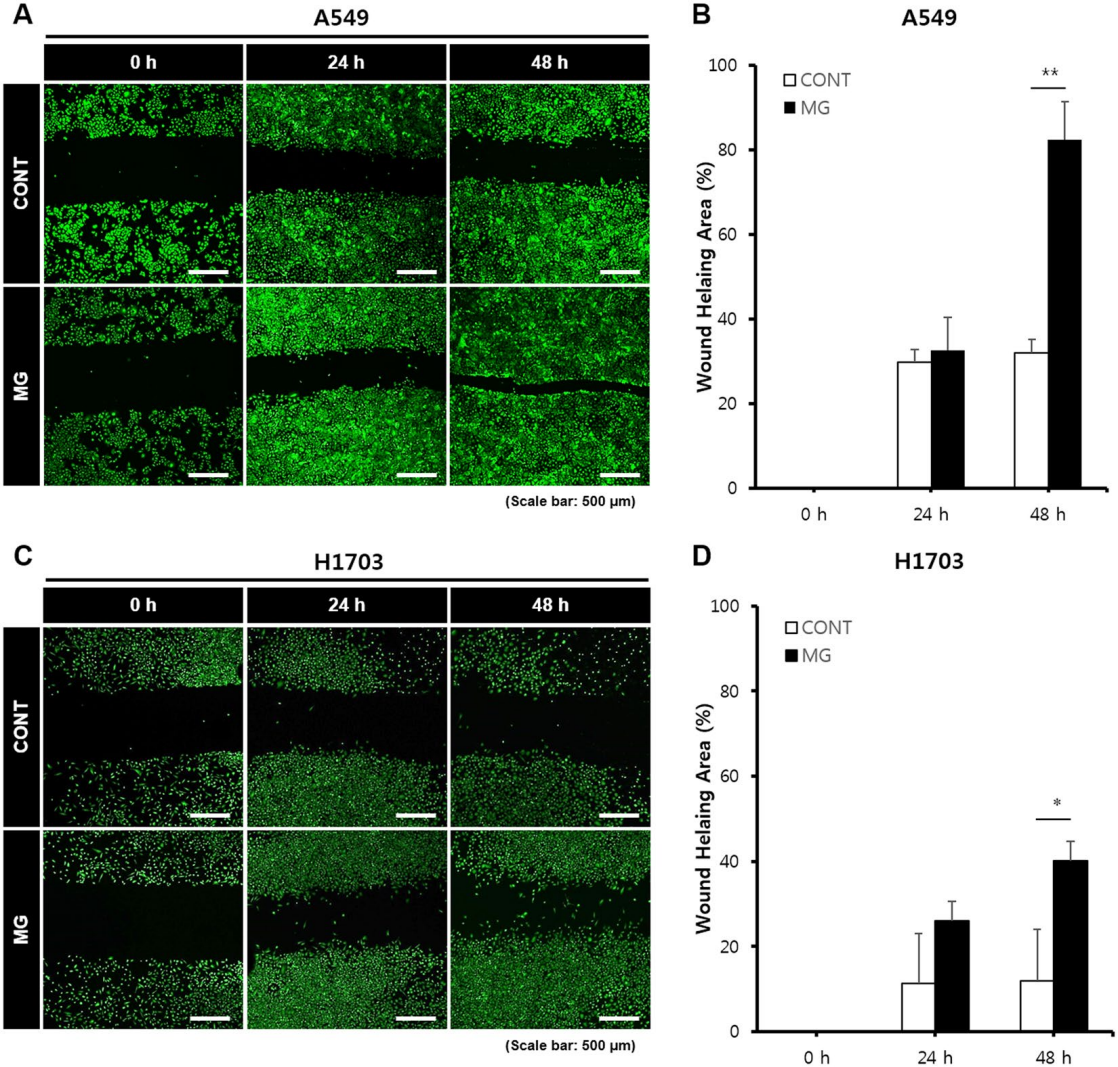
Wound healing analysis of A549 and H1703 cells on CONT and MG. Migration potential of A549 cells in wound healing assay (**A**). Calculated wound healing areas in A549 cells (**B**). Migration potential of H1703 cells in wound healing assay (**C**). Calculated wound healing areas in H1703 (**D**) (*p < 0.05, **p < 0.01).

**Figure 4:**
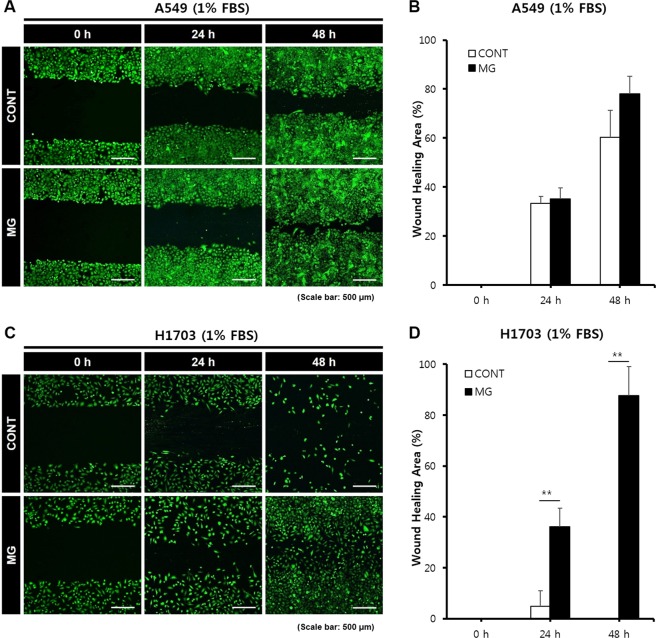
Wound healing analysis of A549 and H1703 at a low cell proliferation condition (1% FBS condition) on CONT and MG. Migration potential of A549 cells in wound healing assay (**A**). Calculated wound healing areas in A549 cells (**B**). Migration potential of H1703 cells in wound healing assay (**C**). Calculated wound healing areas in H1703 (**D**) (**p < 0.01).

To investigate whether microgravity affects the migration potential of A549 and H1703 cells, a wound healing assay was conducted. The wound healing rate of the MG was significantly higher than that of the CONT (Fig. 3). For A549 cells in the MG, after 24 h, the width of the scratched line was reduced by 32%; after 48 h, 82% of the scratched areas was covered with new A549 cells. Although the cell density in the CONT was increased, the migration rate was slower than that in the MG. These results are shown in Fig. 3B. The migration rate of H1703 cells in the MG was higher than that in the CONT. H1703 cells in the CONT and MG showed slower migration rates and lower cell densities than A549 cells (Fig. 3C,D). In order to observe the cell migration while reducing the effect of cell proliferation, we repeated the wound healing assay at 1% FBS (fetal bovine serum) condition. As expected, the wound healing rate in the MG was significantly higher than that in the CONT (Fig. 4). Especially, H1703 of normal gravity (CONT) showed extremely low cell viability and inactive wound healing (Fig. 4C,D).

### Effects of microgravity on gene expression in lung cancer cells

**Figure 5:**
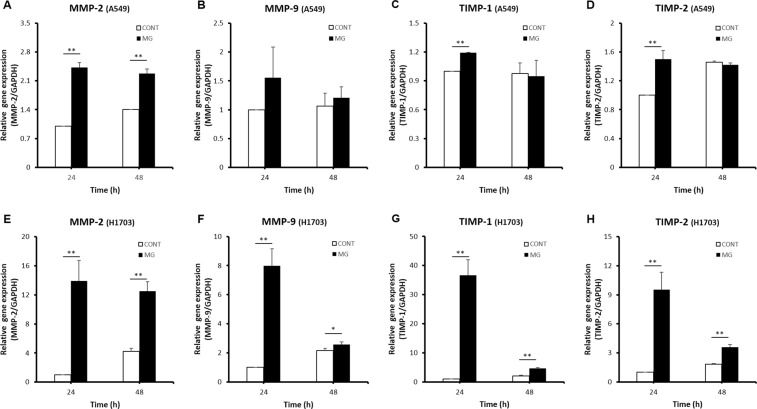
Comparison of expression levels of cell migration related genes between CONT and MG. Relative gene expression levels of *MMP-2* (**A**), *MMP-9* (**B**), *TIMP-1* (**C**), and *TIMP-2* (**D**) in A549 cells. Relative gene expression levels of *MMP-2* (**E**), *MMP-9* (**F**), *TIMP-1* (**G**), and *TIMP-2* (**H**) in H1703 cells (*p < 0.05, **p < 0.01).

Many genes have been reported to be involved in cancer migration and metastasis. We selected the representative markers of tissue invasion and metastasis, MMP-2 and MMP-9 up-regulate lung cancer migration under microgravity conditions. Additionally, TIMP-1 and TIMP-2, which are major repressor genes of metastasis, can up-regulate metastasis depending on the conditions. When adenocarcinoma A549 cells were exposed to the microgravity environment, MMP-2, MMP-9, TIMP-1, and TIMP-2 showed higher expression in the MG than in the CONT at 24 h (Fig. 5A–D). After 48 h, MMP-2 and MMP-9 expression levels in the MG were higher than those in the CONT. In addition, the difference in TIMP-1 and TIMP-2 expression between the MG and CONT was only 2%. Namely, under microgravity, MMP-2 and MMP-9, which up-regulate cell migration, were increased and TIMP-2 and TIMP-9 were highly expressed to counteract MMP gene expression. The expression of MMP-2 and MMP-9 and their counteracting genes TIMP-1 and TIMP-2 gradually decreased over time.

In squamous cell carcinoma H1703 cells, at 24 h, MMP-2, MMP-9, TIMP-1, and TIMP-2 in the MG showed higher expression than in the CONT (Fig. 5E–H). The expression levels of all genes in the MG were still higher than in the CONT at 48 h, although the differences were decreased. Interestingly, TIMP-1 gene expression in the MG was 37-fold higher than that in the CONT at 24 h (Fig. 5G). The difference in TIMP-1 expression between A549 and H1703 cells under the same microgravity conditions demonstrates that these cells have different migration and metastasis potentials, even in the same lung cancers.

### Effects of microgravity on protein expression in lung cancer cells

**Figure 6:**
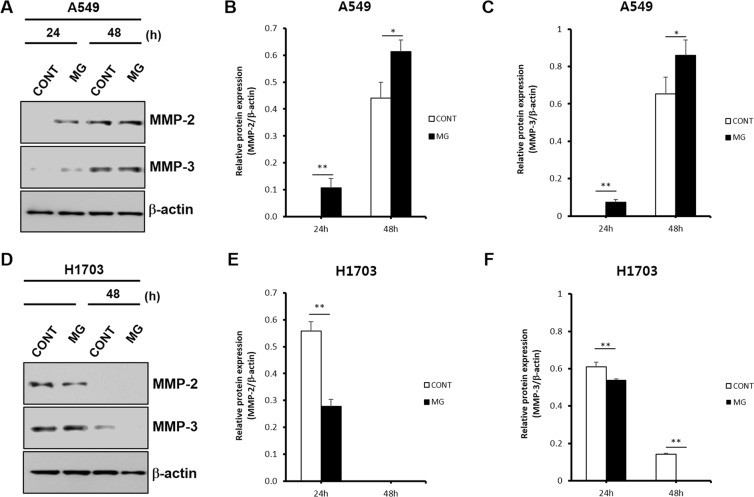
Protein expression evaluation by western blotting of A549 and H1703 on CONT and MG. Comparison of MMP-2 and MMP-3 protein expression between CONT and MG in A549 cells (**A**). Relative MMP-2 protein expression levels of CONT and MG over time in A549 cells (**B**). Relative MMP-3 protein expression levels of CONT and MG over time in A549 cells (**C**). Comparison of MMP-2 and MMP-3 protein expression between CONT and MG in H1703 cells (**D**). Relative protein expression levels of CONT and MG over time in H1703 cells (**E**). Relative protein expression levels of CONT and MG over time in H1703 cells (**F**) (*p < 0.05, **p < 0.01).

To investigate protein expression in lung cancers in a microgravity environment, western blotting was conducted. As a target protein, MMP-2 and MMP-3 were selected based on the RT-PCR results. In A549 cells, MMP-2 protein expression was highly expressed in the MG compared to in the CONT at 24 and 48 h (Fig. 6A,B). However, western blotting for H1703 cells showed different results. MMP-2 protein expression in the MG was lower than that in the CONT and bands in both the MG and CONT had disappeared at 48 h (Fig. 6D,E). In addition, a relative protein expression between MMP-3 and β-actin was similar to that between MMP-2 and β-actin (Fig. 6C,F). It is thought that this decrease in the expression value at the protein level may have made a difference in the migration speed of H1703 and A549.

## Discussion

Although the simulated microgravity created by the RPM does not perfectly reconstitute the zero gravity in space, an RPM can be used to increase the understanding of cell physiology, including cell adhesion, cell cycle, structure, and functions^24–26^. Particularly, in tumor cells, increasing studies have demonstrated that microgravity affects cellular proliferation, apoptosis, invasion and migration, and gene expression^27–29^. In thyroid cancer, microgravity altered differentiation and increased apoptosis^19^. In studies of human breast carcinoma^20^, lung adenocarcinoma^21^, and human glioblastoma^22^, the proliferation and migration abilities between stimulated cancer cells in the microgravity system and non-stimulated cancer cells were compared under 1G condition, which indicated that the proliferation and metastasis of cancer cells were altered by the microgravity environment. However, studies focusing on various lung cancers living in a microgravity environment, rather than carrying out further experiments by taking out cells after microgravity stimulation would not be sufficient. Therefore, in this study, we examined the metastasis of two lung cancer cell lines (A549, H1703) in a floating state using a bioreactor with microgravity.

In the dynamic culture state using the microgravity system, although the microgravity can be adjusted in the system, acceleration occurs when the system starts rotating and the gravitational value periodically changes during the rotating motion. Forces were applied not only to the culture media but also to the membrane culture dishes because of these changes in the microgravity value and acceleration value. This significantly affected cell proliferation over a short incubation time of 24 h. However, when the cells in the culture system were stabilized for 48 h, the proliferation rate of adenocarcinoma A549 was higher in the MG than in the CONT. Supplementation of the simulated microgravity system is necessary to improve initial cell attachment and reduce the effects of external forces.

The wound healing assay results using two types of lung cancer cells clearly demonstrated enhanced cell migration in the MG. Particularly, A549 cells had migrated approximately 700 μm at 48 h because of the enhanced mobility under microgravity conditions. H1703 cell migration was not clearly observed in the control, but a remarkable difference was observed in the MG. A549 showed the ability for rapid migration and proliferation, which was accelerated by microgravity.

Invasive tumor growth and lymph node metastasis are closely related to the proteolytic enzymes MMPs, urokinase-plasminogen activator, and cathepsin B, D, and L^30–34^. Among these, MMP-2 and MMP-9, which are gelatinases in MMPs, play an important role in destruction of the basement membrane and extracellular matrix^35^. In addition, MMP-3 enhances the migratory and invasive abilities of tumor cells^24,36–40^. In our study, as shown by the RT-PCR results, MMP-2 and MMP-9 expression levels in the MG at 24 h were higher than those in the CONT for both A549 and H1703 cells. Although the absolute expression value of MMPs decreased over time, MMP-2 and MMP-9 in the MG showed high expression compared to in the CONT at 48 h. Thus, the microgravity environment up-regulated expression of MMP-2 and MMP-9, which cause cancer cell migration.

Extracellular matrix degradation of MMPs is mainly regulated by TIMPs^41^. In normal tissues, the degradation metabolism of the basement membrane is stabilized by the balance of MMP and TIMP secretion. In tumors, invasion and metastasis can be promoted by an imbalance in these molecules^42^. The role of TIMPs in cancer progression remains unclear. TIMPs appear to inhibit tumor growth and invasion in some tumors, whereas in certain types of malignant tumors high TIMP levels are correlated with more aggressive behavior. Down-regulation of TIMP-1 inhibited cell migration, invasion, and metastatic colonization in lung adenocarcinoma^6^. TIMP-1 promoted the accumulation of cancer associated fibroblasts and cancer progression^43^. Additionally, TIMP-1 promoted vascular endothelial growth factor-induced neovascularization in the retina^44^. Moreover, TIMP-1 promoted liver metastasis by inducing hepatocyte growth factor signaling^45^. Additionally, in renal cell carcinoma, increased expression of MMP-2, MMP-9, TIMP-1, and TIMP-2 was correlated with poor prognostic variables^46^.

In our study, microgravity increased TIMP-1 and TIMP-2 expression as well as MMP-2 and MMP-9 expression in both A549 and H1703 cells at 24 h. This is consistent with the results of other studies showing that expression of TIMPs increased cancer cell migration and metastasis. However, in H1703 cells, the expression level of TIMP-1 at 24 h in the MG was 37-fold higher than that in the CONT, while TIMP-1 expression was not largely different in A549 cells. This may be because overexpressed TIMP-1 under microgravity conditions down-regulated the activities of MMP-2 and MMP-9 in H1703 cells. Thus, A549 cells, with a low TIMP-1 signal difference, showed faster migration than H1703 cells in the wound healing assay. Therefore, it is necessary to carefully observe changes in TIMP-1 levels in addition to MMP-2 and MMP-9 to analyze lung cancer migration in the microgravity environment. All genes evaluated in our study were expressed within 24 h and that meant that the microgravity conditions immediately and effectively influenced the migration of lung cancer cells. Rapid and powerful activation of gene expression was demonstrated in the wound healing assay. Based on the wound healing results in A549 cells, the activated genes increased cell mobility for 48 h.

When the amount of MMP-2 protein synthesized by A549 cells was analyzed by western blotting in the MG, high levels of MMP-2 protein were detected at 24 h, but the protein was not detected in the CONT. At 48 h, MMP-2 protein levels were higher in the MG than in the CONT. Thus, high expression of MMP-2, a key protein in cell migration, in the MG over time demonstrated that microgravity increased the migration of A549 cells, which is consistent with the wound healing assay results. However, in H1703 cells, MMP-2 protein synthesis results was in contrast to the RNA levels because TIMP-1 was overexpressed. At 48 h, protein expression in both the CONT and MG had disappeared and none detection of MMP-2 protein at that time was consistent with the decreased H1703 cell migration rate in the wound healing assay. These results demonstrate that A549 cells migrate more rapidly than H1703 cells. Additionally, NSCLCs show differences in migration and metastasis according to their sub-type, even in the same lung cancers, indicating that the effect of microgravity on migration and metastasis in each cancer is not uniform.

## Conclusion

Simulated microgravity was found to promote cell migration and expression of genes related to different sub-types of NSCLC, which accounts for numerous lung cancers, although the levels of promotion differed by sub-type. However, because of the limitations of the MG conditions and periodic changes in gravity, the MG showed lower cell proliferation values than CONT because of the deterioration of an initial cell adhesion ability. These effects depend on the lung cancer sub-type, and A549 cells showed a higher proliferation rate in the MG over time. The gene expression and protein synthesis results showed that microgravity promoted the migration of non-small cell lung cancer. Additionally, the migration performance of A549 cells was stronger than that of H1703 cells. Based on our results, it is necessary to carefully observe changes in TIMP-1, MMP-2, and MMP-9 levels to analyze the migration of NSCLC. Our results provide a foundation for studies of cancer migration and metastasis.

## Materials and Methods

### Cell culture

The human lung cancer cell lines squamous cell carcinoma (H1703) and adenocarcinoma (A549) cells were purchased from American Type Culture Collection (ATCC, Manassas, VA, USA). The cells were cultured in RPMI 1640 (Gibco, Grand Island, NY, USA) medium containing 10% fetal bovine serum (Gibco) and 100 U/mL of penicillin/streptomycin (Gibco) at 37 °C in a humidified atmosphere containing 5% CO_2_. The medium was changed every 2–3 days. When the cells reached confluence, they were removed from the culture dish using 0.5% trypsin (Gibco)-ethylenediaminetetraacetic acid, centrifuged, and resuspended in RPMI 1640. Next, 10 µL (density of 1 × 10^5^ cells/cm^2^) of the cell suspension was pipetted into the dishes.

### Installation of bioreactor with simulated microgravity

An RPM system (3D clinostat, Shamhantech Inc., Bucheon, Korea), which is an effective system for simulating microgravity on the ground, was used in this study. This system is composed of two rotational axes and the rotation speed of each axis was fixed to 5 rpm (0.523 rad/s). Therefore, the centrifugal accelerations of the system calculated by Eq. (1) were 0.0047 G (edge) to 0 G (center)^9,10^. To create a lung cancer culture environment, 1.7 × 10^5^ cells were seeded onto a polystyrene based culture membrane (density: 0.958 g/cm^3^, SPL Life Sciences Co., Gyeonggi-do, Korea). After preparing an oxygen-permeable soft chamber (SPL Life Sciences Co.), RPMI 1640 medium was added and the chamber was packed with the culture membrane with attached cells. The soft chambers were fixed to the rotating panel of the RPM system, which was placed in a humidified incubator at 37 °C and 5% CO_2_ atmosphere. The system was continuously rotated at 5 rpm for 48 h. The ground group was cultured under the same conditions as the experimental group, but without microgravity.

1 $$\,\begin{array}{ll}{{\rm{a}}}_{{\rm{c}}}={r{\rm{\omega }}}^{2}=\frac{{r{\rm{\omega }}}^{2}}{9.8}\,{\rm{G}} & \\ ({{\rm{F}}}_{{\rm{c}}}={{\rm{ma}}}_{{\rm{c}}}={\mathrm{mr}{\rm{\omega }}}^{2}, & 1{\rm{G}}=9.8({\rm{m}}/{{\rm{s}}}^{2}))\end{array}$$

Where a_c_ is the centripetal acceleration generated by rotating system (m/s^2^), ω is the angular velocity (rad/s), r is the distance of the sample to the center of rotation (m), G is acceleration of gravity, F_c_ is the experienced centripetal force (N) and m is mass (kg).

### Cell viability assays

A cell counting kit-8 (CCK-8, Dojindo Laboratory, Kumamoto, Japan) was used to measure cell proliferation. Briefly, CCK-8 solution and serum-free RPMI 1640 were mixed at a ratio of 1:10. After the cell-seeded membrane in the soft chamber was moved into a new multi-well plate, serum-free RPMI 1640 containing CCK-8 solution was added. After 2 h in a humidified 5% CO_2_ atmosphere, cell proliferation was determined by measuring the absorbance at 450 nm using a microplate reader (Thermo Scientific, Waltham, MA, USA).

### Wound healing assay

A wound healing assay was carried out to evaluate the migration potential of two lung cancer cell lines (A549 and H1703). A549 and H1703 cells were initially seeded on the culture membrane at a density of 1.7 × 10^5^ cells/dish. After the cells were adhered to the membrane for 16 h, a straight scratch was made on the cell monolayer using a sterile 200-µL pipette tip. The scratched areas were immediately and gently rinsed twice with RPMI 1640. After the scratched samples were packed into the soft chamber, the cells subjected to microgravity (MG) was incubated in a simulated microgravity environment in a humidified incubator at 37 °C under a 5% CO_2_ atmosphere for 24 and 48 h. Cells subjected to ground condition (CONT) was incubated in the same environment as the MG group, except without microgravity. After an incubation, wound healing images were acquired using a computer connected microscope. Wound sizes were analyzed using the equation ((wound healing areas/cell-free area of the initial scratch) × 100%).

### Determination of mRNA levels

For quantitative real-time PCR assays, total cellular RNA was extracted from the cells using Trizol Reagent (Invitrogen, Carlsbad, CA, USA) according to the manufacturer’s instructions. Reverse transcription was performed using the iScript™ cDNA synthesis kit (Bio-Rad, Hercules, CA, USA) with 2 µg of total RNA in a 40-µL reaction volume. For real-time PCR analysis, primers for the matrix metalloprotease (MMP)-2, MMP-3, MMP-9, tissue inhibitor of metalloprotease (TIMP)-1, and TIMP-2 genes were designed as previously reported^47,48^. Quantitative real-time PCR was performed with SsoFast EvaGreen supermix using the iQ5 Real-Time PCR Detection System (Bio-Rad). Glyceraldehyde 3-phosphate dehydrogenase (GAPDH), which was shown to be unaffected by exposure to the RPM, was used as an internal control for normalization. Each reaction was performed in duplicate in three independent experiments.

### Western blot analysis

Proteins were extracted from A549 and H1703 cells in the MG group and CONT group, harvested, and lysed on ice for 20 min in RIPA lysis buffer containing protease inhibitor cocktails. The lysis solution was centrifuged at 12,000 rpm for 20 min at 4 °C to remove cellular debris. The supernatants were collected, and the protein concentration was determined using the Bradford method (Bio-Rad). Next, 20 µg of protein was separated on a 12% SDS-PAGE gel and then transferred onto polyvinylidene difluoride membrane (Millipore, Billerica, MA, USA). The blots were blocked with 5% skim milk and incubated overnight at 4 °C with MMP-2 and MMP-3 (Abcam, Cambridge, UK) or β-actin (Sigma, St. Louis, MO, USA) antibodies at a dilution of 1:1000. After washing with Tris-buffered saline containing 0.05% Tween-20, the blots were incubated for 1 h at room temperature with anti-rabbit IgG or anti-mouse IgG horseradish peroxidase-linked secondary antibodies (1:2000). Protein bands were visualized using ECL plus (Thermo Scientific) detection. β-actin was used as the loading control.

### Statistical analysis

All experiments were conducted in triplicate and representative or average data are presented unless otherwise stated. The data were presented as the mean ± standard deviation. Single-factor analysis of variance with Tukey’s multiple comparison test was performed to determine statistical significance (*p < 0.05, **p < 0.01).

## Supplementary information

Supplementary Information
