# Supplementary material for: Simulated microgravity with floating environment promotes migration of non-small cell lung cancers
Source: Sci Rep. 2019 Oct 10;9:14553. doi: 10.1038/s41598-019-50736-6 (PMC6787256; doi:10.1038/s41598-019-50736-6)
Supplement: Supplementary file 1 — Supplementary Information [file 41598_2019_50736_MOESM1_ESM.pdf]

Supplementary Information for

Simulated microgravity with floating environment promotes migration of non-small cell lung cancers

Chi Bum Ahn [1, *], Ji-Hyun Lee [1, *], Dae Geun Han [2], Hyun-Wook Kang [3], Sung-Ho Lee [4], Jae-Ik Lee [5], Kuk Hui Son [5, †], Jin Woo Lee [1, 2, †]

[1] Department of Molecular Medicine, College of Medicine, Gachon University, Incheon, Republic of Korea

[2] Department of Health Sciences and Technology, GAIHST, Gachon University, Incheon, Republic of Korea

[3] Department of Biomedical Engineering, School of Life Sciences, Ulsan National Institute of Science and Technology, Ulsan, Republic of Korea

[4] Department of Thoracic and Cardiovascular Surgery, Korea University Medical College, Korea University, Seoul, Republic of Korea

[5] Department of Thoracic and Cardiovascular Surgery, Gachon University Gil Medical Center, School of Medicine, Gachon University, Incheon, Republic of Korea

[*] These authors contributed equally to this work.

[†] Corresponding authors.

Please address all correspondence to Professor Kuk Hui Son and Jin Woo Lee.

Supplementary figure

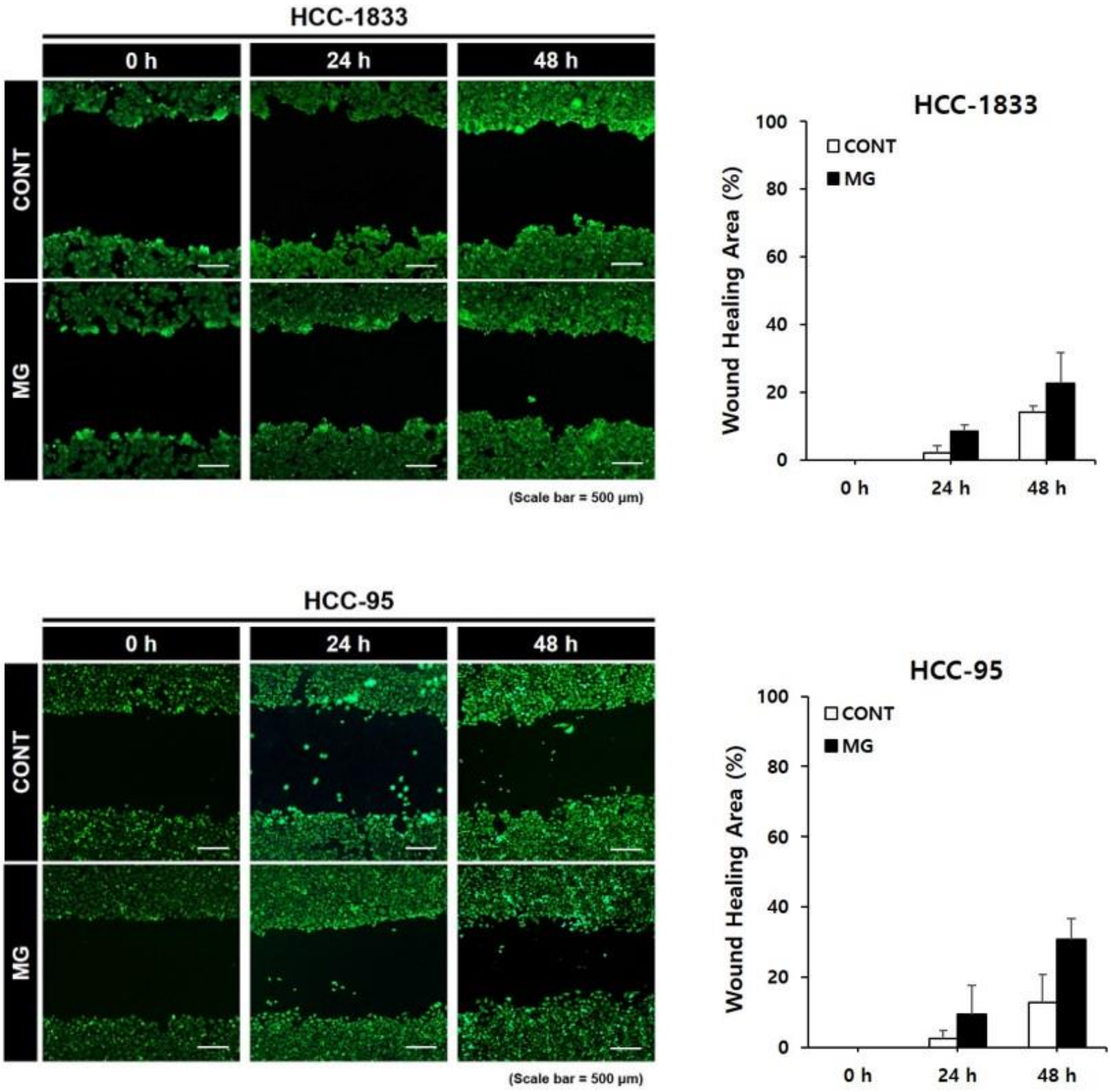

Supplementary figure 1. Wound healing analysis of HCC-1833 (adenocarcinoma) and HCC-95 (squamous cell carcinoma) at a low cell proliferation condition (1% FBS condition) on CONT and MG (**$p < 0.01$).

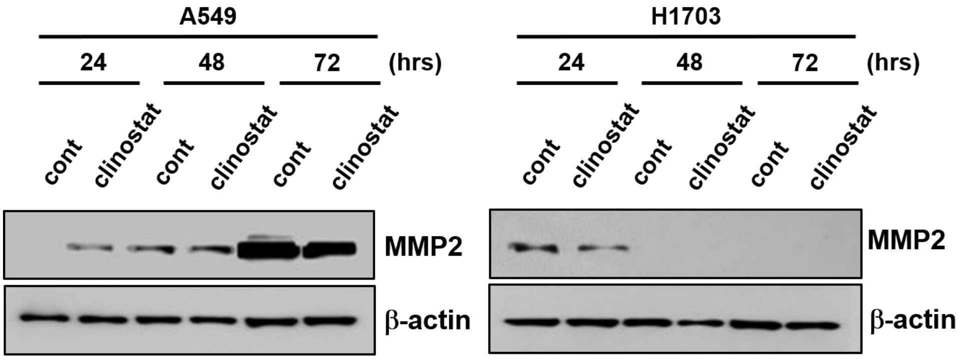

Supplementary figure 2. Long term comparison of MMP-2 protein expression between CONT and MG in A549/H1703 cells.

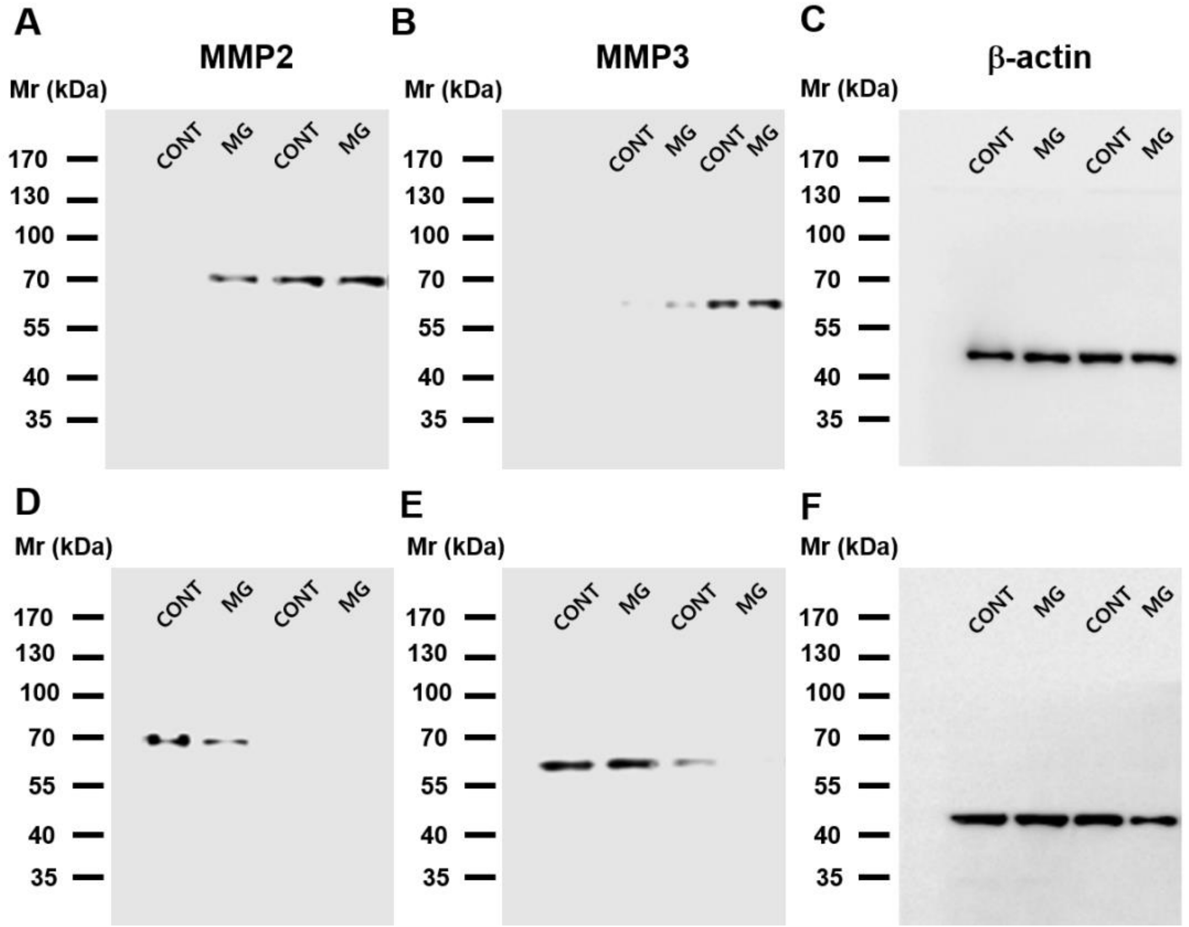

Supplementary figure 3. Original gel images by western blotting. MMP-2 (A), MMP-3 (B) and β-actin (C) protein expressions between CONT and MG in A549 cells. . MMP-2 (D), MMP-3 (E) and β-actin (F) protein expressions between CONT and MG in H1703 cells.
